# Supplementary figures and images for: Dyrk1a gene dosage in glutamatergic neurons has key effects in cognitive deficits observed in mouse models of MRD7 and Down syndrome
Source: PLoS Genet. 2021 Sep 29;17(9):e1009777. doi: 10.1371/journal.pgen.1009777 (PMC8480849; doi:10.1371/journal.pgen.1009777)

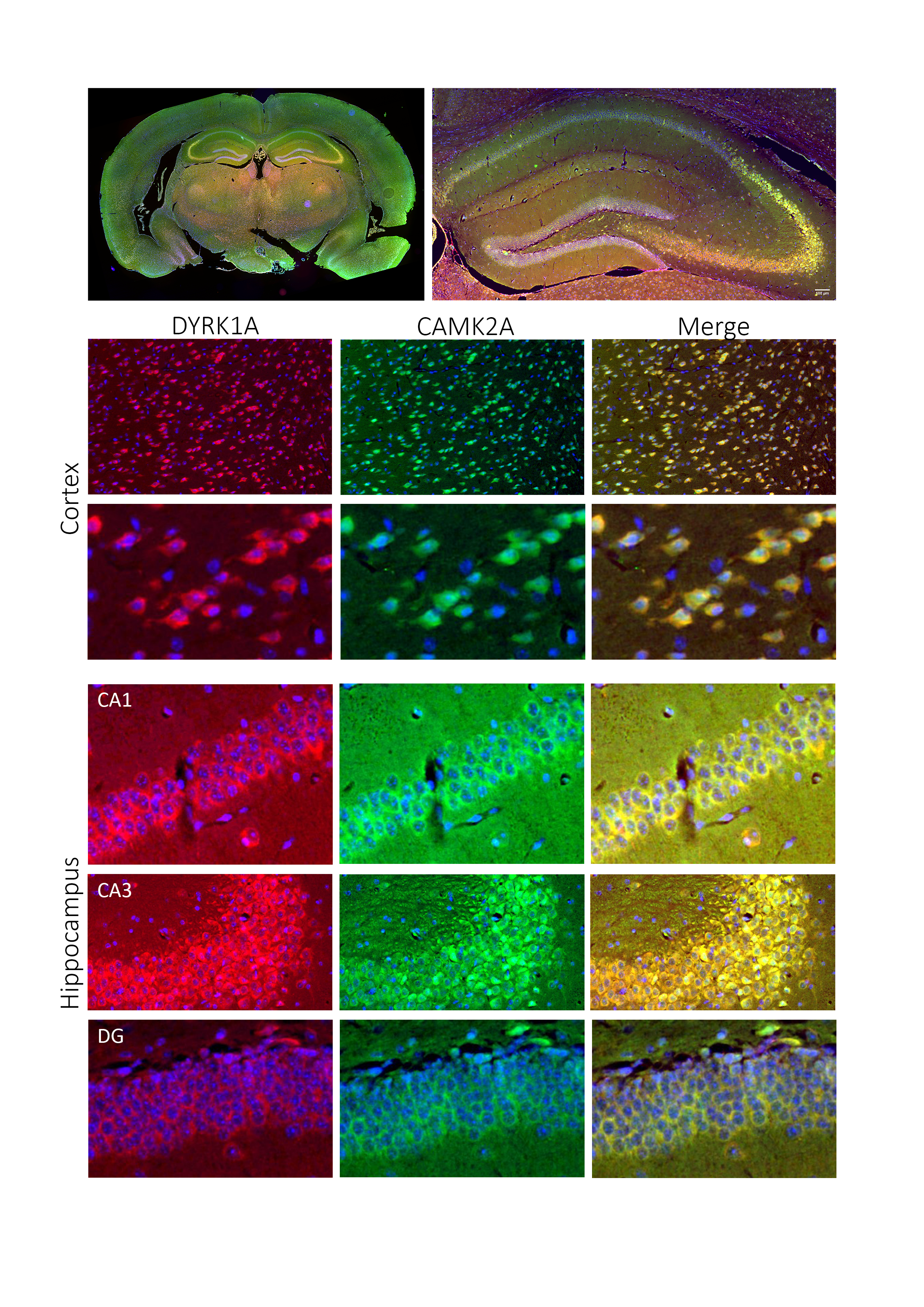

Supplement: S1 Fig — (TIF) [file pgen.1009777.s012.tif]

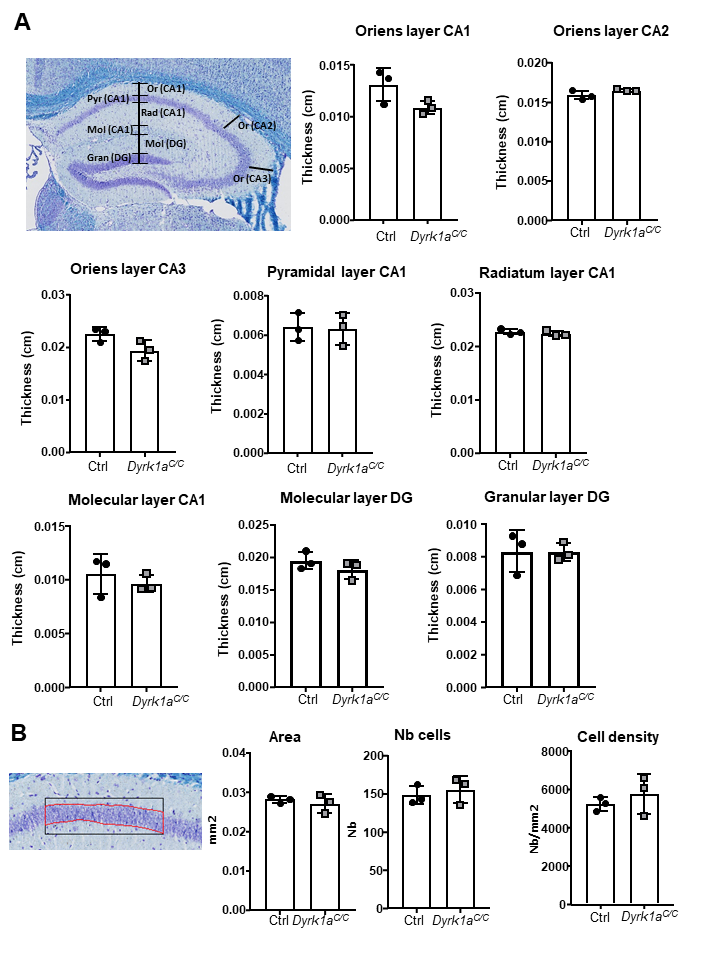

Supplement: S2 Fig — (A) Representative coronal section of hippocampus at Bregma -1.5 stained with cresyl violet and luxol blue that were used for measurements (Magnification 20X) and dot plots indicating the thickness of the different cellular and molecular layers. (B) Enlarged image of the CA1 showing the selected area made for counting the number of cells within the CA1 and dot plots for the area of the CA1, the number of cells within this area and the cell density. Data are presented as point plots with mean ± SD (n = 3 females aged 3 months per genotype). Pyr: pyramidal layer, Mol: molecular layer, Gran: granular layer, Or: oriens layer, Rad: radiatum layer. (TIF) [file pgen.1009777.s013.TIF]

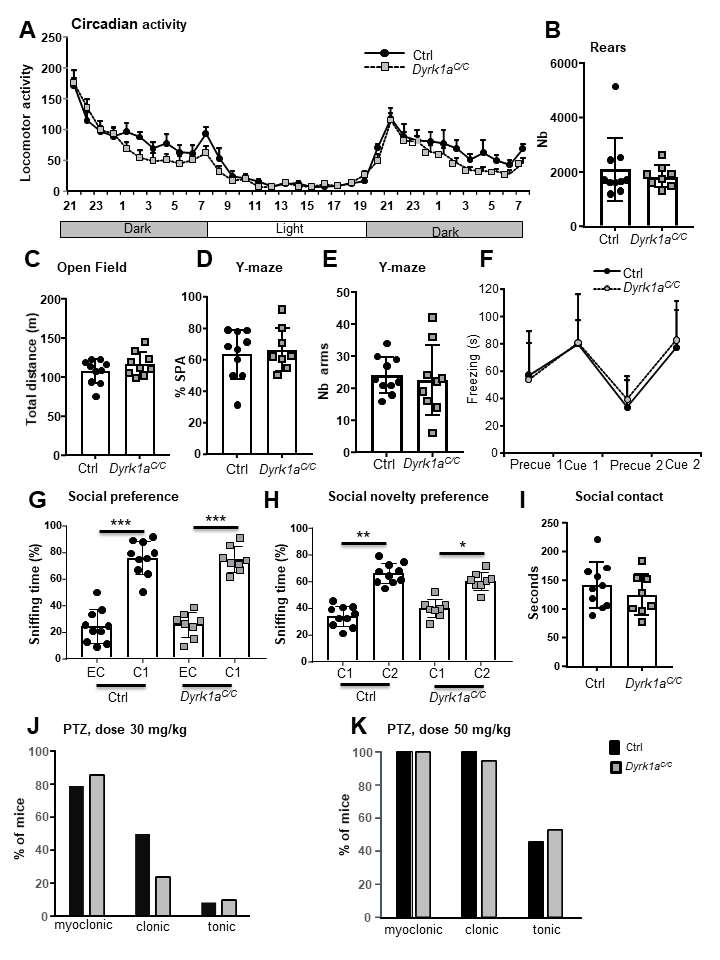

Supplement: S3 Fig — (A-B) Effects of Dyrk1a inactivation on circadian activity. Locomotor activity during circadian analysis was comparable between Dyrk1aC/C and control mice through the light/dark cycle. Numbers on the Y-axis represent the hours. Data are presented as mean ± SEM for each hour. (B) Dot plot of the total number of rears registered during the whole 35H-period of circadian analysis. (C) The total distance travelled during 30 min within the OF was comparable between genotypes. (D) Working memory assessed by percentage of spontaneous alternation within the arms of the Y maze was not impacted by inactivation of Dyrk1a in Dyrk1aC/C mice. (E) The locomotor activity assessed by the number of arm entries was also similar between the two genotypes. (F) In the fear conditioning test, the baseline level of immobility (precue 1 and precue 2) and the cued freezing performances (cue 1 and cue 2) in a new context were comparable between genotypes. (G-I) Assessment of social behavior in the Crawley three-chamber test shows that both genotypes spend more time exploring the cage containing a congener than the empty cage (G; paired t-test congener vs empty cage: ctrl, ***p<0.001 and Dyrk1aC/C ***p<0.001) and exploring the novel than familiar congener (H; paired t-test new congener vs familiar congener: ctrl, **p = 0.002 and Dyrk1aC/C *p = 0.019). (I) Social contact assessed by measuring to time spent sniffing both congeners during the test for novelty preference was similar between mutant and control mice. A-I: tests were done on males (1.5–3.5 months old depending on the test with animals aged ± 3 weeks), n = 8–10 per genotype. Data are presented with mean±SD. (J-K) Epileptic susceptibility was tested with the injection of two doses of PTZ in 6 months old male mice. Percentage of mice reaching myoclonic, clonic and tonic seizure stage were similar between the two genotypes at dose 30 mg/kg body weight (J; n = 25 ctrl and n = 20 Dyrk1aC/C mice) and 50 mg/kg body weight (K; n = 22 ctrl and [file pgen.1009777.s014.TIF]

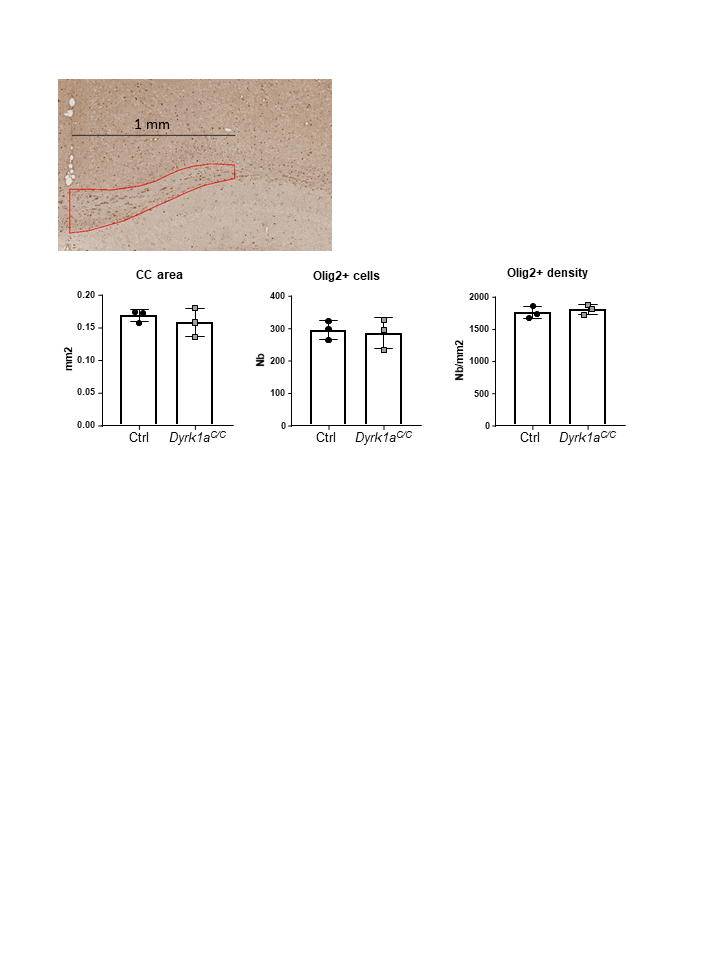

Supplement: S4 Fig — A) distance of 1 mm was measured and the underneath corpus callosum was selected. The cc area as well as the number of OLIG2+ cells and the OLIG2+ cell density did not differ between Dyrk1aC/C and control animals. Data are presented as point plots with mean ± SD (each dot represents the mean count of 3 serial sections). (TIF) [file pgen.1009777.s015.TIF]

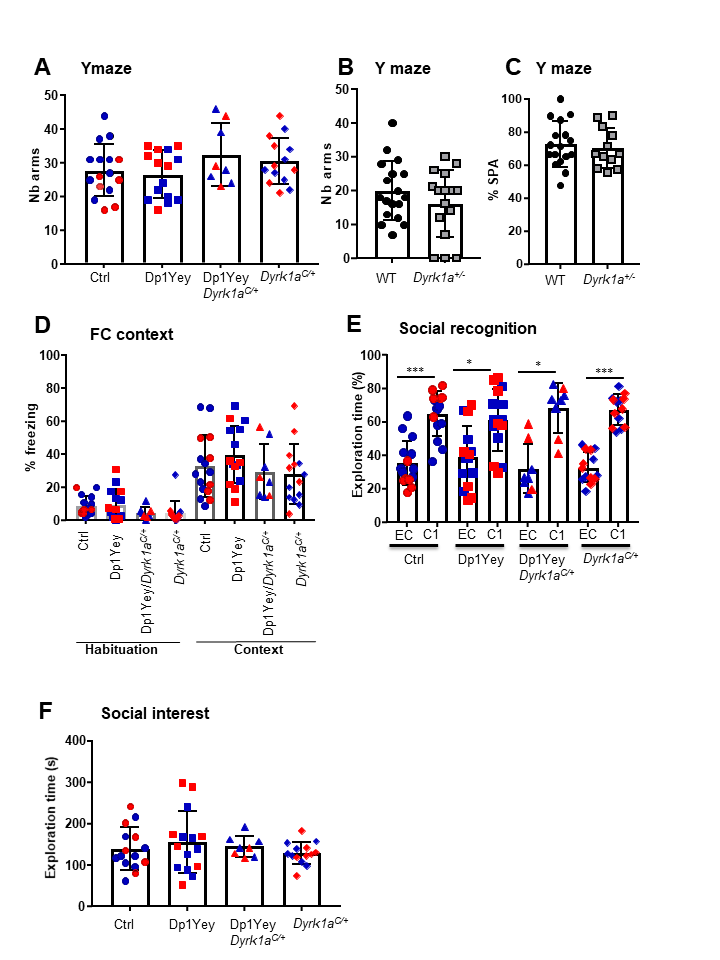

Supplement: S5 Fig — (A) The locomotor activity assessed by the number of arm entries in the Y maze was similar between control, Dp1Yey, Dp1Yey/Dyrk1aC/+ and Dyrk1aC/+ genotypes. (B-C) Activity (B) and working memory (C) was assessed in Dyrk1a full heterozygous knockout (Dyrk1a+/-) mice in the Y maze showing no effect of Dyrk1a haploinsufficiency (only males were analyzed here). (D) In the fear conditioning test, the baseline level of immobility during the habituation period was similar between genotypes and contextual freezing performance in the same environment after conditioning was also comparable between genotypes. (E-F) Assessment of social behavior in the Crawley three-chamber test shows that all genotypes spend more time exploring the cage containing a congener than the empty cage (E; paired t-test congener vs empty cage: ctrl, ***p<0.001; Dp1Yey, *p = 0.03; Dp1Yey/Dyrk1aC/C, *p = 0.01; Dyrk1aC/C, p***p<0.001). Moreover, no difference was found between genotypes in the total time spent sniffing the cage containing a congener (F). Data are represented as point plots with mean ±SD. Males are in blue and females are in red. (TIF) [file pgen.1009777.s016.TIF]

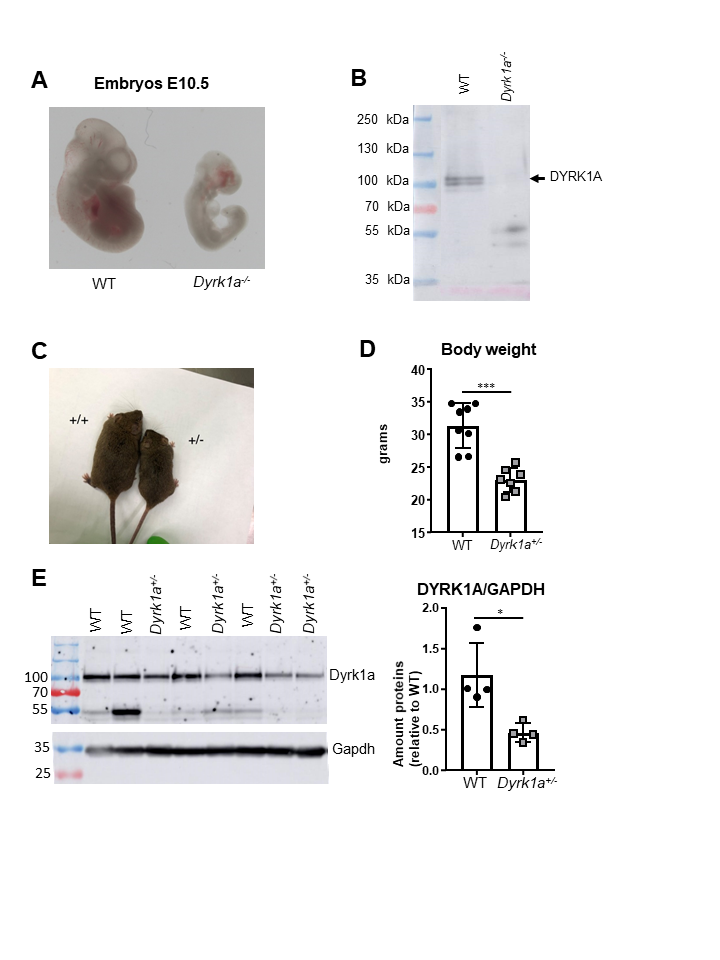

Supplement: S6 Fig — (A) Photo of a wild-type (WT) and a Dyrk1a-/- embryos at E10.5 showing growth retardation of the Dyrk1a knockout animal. (B) Western blot analysis of equivalent amounts of protein extracts from WT and Dyrk1a-/- embryos showing the absence of the Dyrk1a-specific protein at around 100 kDa. A band is seen at around 55kDa, but this is an unspecific band that appears sometimes in our blots as seen also in wild-type protein extracts in panel E. (C) Photo of a 4-months-old WT male and Dyrk1a+/- littermate showing significant body size reduction. (D) Body weight of WT and Dyrk1a+/- twelve weeks old males. (E) Western blot analysis: autoradiographic image and quantification of immunoblots of Dyrk1a protein in the hippocampus of WT and Dyrk1a+/- animals (8 WT and 7 Dyrk1a+/- 12-weeks old males). Band intensities were estimated using ImageJ and normalized against the loading control Gapdh (or against total loaded proteins visualized with Ponceau red). Data are presented as point plots with mean ± SD with unpaired Mann-Whitney test, *p<0.05, ***p<0.001. (TIF) [file pgen.1009777.s017.TIF]
