# Supplementary material for: Dyrk1a gene dosage in glutamatergic neurons has key effects in cognitive deficits observed in mouse models of MRD7 and Down syndrome
Source: PLoS Genet. 2021 Sep 29;17(9):e1009777. doi: 10.1371/journal.pgen.1009777 (PMC8480849; doi:10.1371/journal.pgen.1009777)
Supplement: Attachment — Submitted filename: Review_MA.docx [file pgen.1009777.s018.docx]

The manuscript by Brault et al. focusses on the role of DYRK1A kinase in the glutamatergic neurons of the cerebral cortex and hippocampus. *DYRK1A* gene is on chromosome 21 and it is widely expressed in the developing and mature brain. The authors generated a conditional KO mouse designed for the elimination of DYRK1A in the glutamatergic neurons of the postnatal forebrain and assessed the impact of this conditional mutation on brain morphology, cognition and other behaviours associated to MRD7, a rare intellectual disability syndrome caused by loss-of function mutations in the *DYRK1A* gene. In this conditional KOs, they found alterations in the cytoarchitecture of the neocortex and specific cognitive functions. Moreover, they show RNA-seq data suggesting that the lack of DYRK1A in glutamatergic neurons affects the expression of genes involved in synaptic activity and transcription. Using the new conditional *Dyrk1a* mutant they normalize *Dyrk1a* gene-dosage in the glutamatergic neurons of the Dp1Yey mouse, a trisomic model for Down syndrome. Then, they performed several behavioural tests in disomic and trisomic mice with or without the conditional *Dyrk1a* mutation in heterozygosity. Their results show that both haploinsufficiency of *Dyrk1a* and trisomy of Dyrk1a in postnatal glutamatergic neurons have detrimental effects on explicit memory. Finally, they present proteome data indicating that DYRK1A may regulate the activity of glutamatergic synapses.

The study is of interest and some of the results are well explained and are convincing, but others have raised some concerns that the authors need to address by providing new information and experimental data.

Major issues:

1. The quality of the brain images in Figure 1 need to be improved, especially in panel E. The authors should include information in the figure and the legend regarding the area of the neocortex, the age of the animal and, in panel E, the number of animals analysed.

DYRK1A staining in the neocortex is mostly associated to the neuropile (Martí et al., Brain Res 2003), but this do not seem to be the case in the images of the cortex shown in panels A and E, where DYRK1A labelling in the soma of CAMK2+ cells is high. Is DYRK1A staining similar in other cortical regions (e.g. somatosentory and motor cortex)? Did the authors perform control stainings with secondary antibodies?

Cre-recombinase in the Tg(CamK2-Cre)4 is wildly expressed in the postnatal forebrain. The authors should emphasise this point at the beginning of the Results. Do the authors know the onset of Cre-recombinase expression in the hippocampus and neocortex of cKO mice? This information is relevant for the correct interpretation of the results and should be added in the manuscript.

2. Based on morphological alterations of Dyrk1a KO neocortices (thickness and total cell densities; Figure 2) and the similarities of these alterations with those previously observed in Dyrk1a+/-, the authors suggest that DYRK1A has a direct role on late morphological differentiation of glutamatergic neurons. This is an interesting possibility that could be tested by looking in the conditional Dyrk1a KO¡ the morphology of projection cortical neurons after synaptic pruning.

Regarding the data shown in Figure 2. Cell density in the image of the Dyrk1a KO somatosensory cortex (panel F) seems to be decreased rather than increased, in particular in layers II-III. Is this a representative image? The authors should provide magnifications of the images to better illustrate the differences in cell numbers and cell densities between genotypes. In addition, they should label the glutamatergic neurons (using for instance the neuronal marker NeuN) of the adult cerebral cortex to confirm that neuronal densities are indeed augmented in the SSC of Dyrk1a KOs.

3. Differences between the transcriptome of controls and Dyrk1a KOs are based on RNA-seq data obtained from only two animals per genotype. The authors should definitely perform RT-PCRs to confirm the differential expression of immediate early genes and genes involved in neurotransmission in additional animals. Otherwise, this data should be removed.
4. The results of the proteome analysis are interesting but are too short to conclude that DYRK1A in glutamatergic neurons regulates synaptic activity in a gene-dosage dependent manner. The authors should perform additional experiments (measure LTP or at least check by immunoblotting the levels of several of the putative differentially expressed proteins involved in synaptic activity, including CAMK2) to support this conclusion.

The co-IPs shown in figure 5 suggest that DYRK1A and CAMK2 are part of the same protein complex. Is SYN1 also part of this complex? On the other hand, DYRK1A seems to interact with proteins of postsynaptic densities. Is there any evidence that DYRK1A is in the presynaptic part and PSD of excitatory synapses?

The title of this section “Proteomic analysis confirms…..” is an overstatement, I suggest to change it.

Minor issues:

1. According to ORPHANET, the name for MRD7 (Mental Retardation Dominant 7) is DYRK1A-intellectual disability syndrome. This syndrome is also named “DYRK1A haploinsufficiency syndrome”. I suggest to use one of these two names instead of MRD7. The authors should definitely indicate the ORPHA numbers of the syndrome. Additionally, they should add in the Introduction information regarding the type of mutations that are causing the syndrome and, in References, two papers by van Bon et al., one describing the first case with an intragenic deletion in *DYRK1A* genes (Clin Genet. 2011) and, the second one, the main clinical features associated to the syndrome (Molecular Psychiatry 2015).
2. There is information in the Introduction regarding the molecular functions of DYRK1A that is not relevant for the paper and should be eliminated or at least shortened (paragraph lines 79 to 88). Instead, the description of the neurological deficits and behaviours associated to MRD7 is too short.
3. Haploinsufficient Dyrk1a+/- mice show epileptic activity and susceptibility to hyperthermia-induced seizures as well as deficits in social communication (Raveau et al. Neurobiol Dis 2018 and Arranz et al. Neurobiol Dis. 2019). The authors should consider these two works for the interpretation and discussion of their data (deficits in social behaviour and lack of response to PTZ in Dyrk1a KOs) in the context of MRD7.
4. Please indicate the age of the animals used in each experiment/behavioural test in the figure and/or the figure legend. In Fig. 3, also indicate the sex of the animals tested and for clarity, use the same colour code than in Fig. 4.
