# Supplementary material for: Dyrk1a gene dosage in glutamatergic neurons has key effects in cognitive deficits observed in mouse models of MRD7 and Down syndrome
Source: PLoS Genet. 2021 Sep 29;17(9):e1009777. doi: 10.1371/journal.pgen.1009777 (PMC8480849; doi:10.1371/journal.pgen.1009777)
Supplement: Attachment — Submitted filename: 1b. Responses to the reviewers F.docx [file pgen.1009777.s019.docx]

Reviewer's Responses to Questions

**Comments to the Authors:
Please note here if the review is uploaded as an attachment.**

Reviewer #1: The manuscript by Brault et al., “Dyrk1a gene dosage in glutamatergic neurons has key effects in cognitive deficits observed in mouse models of MRD7 and Down syndrome” is a state of the art study to understand the specific effects of Dyrk1a dosage imbalance in the hippocampus with regards to cognitive deficits. This study examines mouse models with 0, 1, 2, and 3 copies of Dyrk1a specific to the glutamatergic neurons of the hippocampus and cortex, and how the dosage imbalance of Dyrk1a affects learning and memory. These data are important to understanding how DYRK1A dosage imbalance causes phenotypes associated with MRD7 (reduced dosage of DYRK1A) and Down syndrome (increased dosage of DYRK1A). There are a number of issues, however, with the study and methodology that need to be clarified so the results may be analyzed in the proper context of the materials and methods used.

Major concerns:
1. What is the genetic background of the Dyrk1aC/C, Dyrk1a+/-, Dyrk1aCamk2aCre/+, Dp1Yey, Dp1Yey/Dyrk1aCamk2aCre/+, Dyrk1acKO/+, and transgenic Tg(Camk2-Cre)4Gsc mice? Are Dyrk1aCamk2aCre/+, Dp1Yey, and Dp1Yey/Dyrk1aCamk2aCre/+, and Dyrk1acKO/+ all littermates with a similar background (Results beginning in line 294?) What were the breeding schemes used to generate these mice? Is it valid to compare Dyrk1aC/C and Dyrk1aC/+ mice if they are not on the same genetic background (lines 341-342)? Likewise, is it valid to compare results from Dyrk1aC/C and Dyrk1a+/- mice if they are not on the same genetic background (lines 401-407)?

YH> We acknowledge that this information was not detailed in the previous manuscript, and we thank the reviewer for pointing this. The description of the different genotypes with their genetic background has been added in a table (suppl. Table S7). The breeding schemes has been included to Mat and Meth (Page 27, lines 614-627). Dyrk1aC/C on C57BL/6N background, and Dyrk1aC/+ mice have a mix B6J/B6N due to the crossing with Dp1Yey animals.

We agree that the genetic background can influence the expression and penetrance of phenotypes. Thus, we have changed the sentences in the discussion indicating the genetic background (Page 20, line 443):*“Dyrk1a^C/C^* mice are viable and present a mild microcephaly with a 10% brain weight reduction and a principal impact on cortical thickness in the pure B6N genetic background. In comparison, *Dyrk1a^+/-^* mice from a mixed B6/129OLA genetic background showed 30% brain reduction and the cortical size reduction was also associated with increased cell density, as described in *Dyrk1a^+/-^* [34; 26], suggesting a reduction of neuronal processes as observed in the neocortex of *Dyrk1a^+/-^* mice [19, 26, 54].”

2. Why is it necessary to have a full knockout of Dyr1ka in the glutamatergic neurons to see the cognitive phenotypes whereas humans with a single mutation in DYRK1A show these phenotypes?

YH> We agree with the reviewer that a full knockout of Dyrk1a in the glutamatergic neurons does not reflect the single mutation in DYRK1A in human with the MRD7/haploinsufficiency of DYRK1A. As stated in the text: Page 7, lines145-146, the full KO was done “*To better understand the function of DYRK1A in adult glutamatergic neurons, circumventing the effect of Dyrk1a on brain development.*”. This of course does not mimic MRD7, which was later analysed with the *Dyrk1a^C/+^* animals but the analysis of the full knockout of *Dyrk1a* in the glutamatergic neurons will give a deeper insight to the function of *Dyrk1a* in those MRD7 neurons.

3. Please outline the numbers of male and female mice in each experiment and whether the tissues tested came from male or female mice. This would be useful information to have in the materials and methods or all figure legends or both. In Figure 4 and supplementary Figure 4, the mice are colored by sex and this would be helpful in all figures. It was noted, by looking at the separation of colors, especially in 4E of the text and 4E of the supplementary figures, that there could have been some sex-specific differences in NOR retention and social recognition, respectively. Are there enough mice in each experiment to accurately test for sex-specific differences in each phenotype (Lines 759 and 773-774 and 897-898)? Not properly analyzing potential differences between sexes in each experiment is a significant shortcoming of the manuscript.

YH> Number, sex and age of the animals used for histological and molecular analyses have been added in Material and Methods. For behavioral analyses, we added the suppl. table S10.

Mice are not colored by sex in other figures because only males were used for *Dyrk1a^C/C^* behavioral analysis (information added to Material and Methods). As stated in Material and Methods, the Dp1Yey allele is only transmitted at 30% in live individuals thus, it was difficult to obtain Dp1Yey*; Dyrk1a^C/+^* and we decided to pool males and females for the analysis to have enough animals for statistical analysis. Hence, we focused on phenotypes with no strong sex effect.

We were confident in pooling both males and females for behavioral analysis as there are no data suggesting a sex effect in ID observed in DS persons and behavioural analyses done on both males and females in the other Ts65Dn DS model (Faizi et al. 2011, PMID: 21527343) (locomotor activity, spontaneous alternation, water maze, and contextual fear conditioning) showed no difference between males and females. In the Crawley social recognition 3-chamber test, a normal preference of Ts65Dn mice for the cage containing a mouse to the empty cage was found for both males and females. Generating and analyzing new animals to carry a sex-difference analysis with more animals would take us about a year of work months. It represents a new project far beyond the conclusions drawn here. Accordingly, we pointed the limitation in the Material and Methods by adding:

Page 34, lines 801-: “*Due to the difficulty to obtain Dp1Yey; Dyrk1a^C/+^ mice* *(three alleles combination and subfertility of the Dp1Yey line with only around 30% transmission of the Dp1Yey allele) and according to the fact that no sex effect was observed in behavior tests done another trisomic model [100], both males and females were pooled for statistical analysis.”*

4. Please state the age of mice used in each experiment. Why were each of these ages chosen? For example, why was the transcriptional profiling done at P30? What was the age of the mice for the morphometric analyses? Is it valid to compare mice that are between 2.5 and 7 months of age on behavioral tests (lines 754 and 755)?

YH> We did not compare animals aged 2.5 months to animals aged 7 months. We apologize for the sentence that was not clear and rephrased it. We meant that different tests were done on animals between 2.5 month and up to 7 months (see sup table S10). For all the tests (molecular, histological and behavioural), animals compared together were littermates.

For histological and molecular analyses, we added the age and sex of the animals in the Material and Methods.

Transcriptome profiling was done at P30, a young adult age, when the brain is fully developed and mature before the phenotyping to avoid any confounding effect due to behaviour testing.

We added this information in both Results (page 12, lines 278-281: “*To unravel the potential molecular mechanisms underlying the learning defects of Dyrk1a^C/C^ mice, we performed genome-wide transcriptional profiling (RNA-seq) of Dyrk1a^C/C^ and control mice in the hippocampus at postnatal day 30, as the brain is fully developed and mature at this stage*”) and Material and Methods, (page 31, lines 728-729: “*Total RNA was Trizol-extracted from two wild-type and two Dyrk1a^C/C^ frozen P30 young adult male hippocampi, stage at which the brain is fully developed.”* ).

5. In Figure 1A, please show the regions that the inset pictures came from on the hippocampus and cortex. In the hippocampus in Figure 1A, it appears that both DYRK1A and CAMK2A are expressed outside or around the neurons and not in the neurons as compared to the cortex. Please comment on this.

YH> We have redrawn the figure 1 to indicate where the insets are located. Figure 1A has been improved as also asked by reviewer 3 and additional information are now in supplementary Figure 1. We have added the DAPI staining to the images to better see that DYRK1A and CAMK2A signals are mainly perinuclear. Our immunostaining for DYRK1A is similar to that shown by Altafaj et al. 2013, figure 3B) (PMID: 23220201). To convince you about this, we provide you bellow with another immunostaining of DYRK1A (red) with GAD65/67 (green) which is expressed around the glutamatergic pyramidal neurons of the CA1. As you can see in this picture, DYRK1A is present within the neurons while GAD65/67 locates around them.

6. The interpretation of morphometric and cellular changes is severely hampered by the methods used to quantify these parameters. In the manuscript these results are reported on a chosen area or cells counted within a chosen area. Stereological methodology should be used to calculate volumes and total numbers of cells in structures, as is the standard. The manuscript should compare volumes of the parts of the hippocampus and total cell number within the hippocampus. In line 170, the manuscript says that “the total number of neurons is unchanged.” This is incorrect. The total number of neurons was not quantified, only a density of neurons in an area that was not detailed as to how it was chosen. How was a particular area (counting frame) chosen for cell counting or other quantification?

YH> We agree with the limitation of the study, and we have rephrased this in our manuscript.

We added in the paragraph “Morphometric analysis and cell counting” in Material and Methods, page 31, lines 723-755:“*It should be noted that cell counts were not acquired using randomly sampled unbiased stereological standards and therefore cannot necessarily be extrapolated to the entire cortex and hippocampus*.” We modified the sentence page 8, lines 180-181 in the results:

*“We counted cells within a specific frame in the middle of the CA1, but found no difference in cell density* *(Supplementary figure 2B).”* We specified why we chose areas for cell counting in Results:

*Page 8 line 168 “We selected sections at Bregma -1.5 to have the striatum and the cortex for morphometric analysis, as those two structures are known to be impacted in by Dyrk1a gene dosage change.”*

*Page 8 line 178 “Within the hippocampus, the dorsal CA1 region has a central role in episodic-like memory formation and was shown to consolidate memory during object novelty discrimination.”*

*Page 8 lines 182-184 “Dyrk1a was reported to impact cortical thickness especially at the level of the somatosensory cortex, with no impact on overall cell density.”*

If the number of neurons were not counted, how can the statement in lines 260-264 be made? Numbers of neurons were not counted, only density. This is a significant deficiency in the current manuscript and needs to be addressed or the present methodology better described and justified.

YH> We removed statement in lines 260-264. We based our analysis on the published method from Mikhaleva et al., 2016. Histomorphological Phenotyping of the Adult Mouse Brain. Curr Protoc Mouse Biol.6(3): 307-32. (ref 98). We further explained our morphometric analysis in Material and Methods: page 30 lines 707-710 *“Coronal sections for morphometric analysis and cell counting were carefully selected using specific anatomical landmarks that enables to precisely select slides that are at the same antero-posterior level (sections showing upper and lower arms of the dentate gyrus of the same length).”*

Minor concerns:
1. The statement “Down syndrome (DS; Trisomy 21), is the first genetic cause of mental retardation” (Lines 71 and 72) is confusing. Perhaps another word in place of “first” such as “major” or “leading?”

YH> We changed to “leading” page 3, line 73: “*Down syndrome (DS; Trisomy 21), is the leading genetic cause of intellectual disability*”.

2. The concept of a “Down syndrome Critical Region” (lines 73-74) has been disproven many times. It is not “a critical region…associated with DS features including mental retardation.” Please remove this outdated concept from the manuscript.

YH> Sentence has been removed and replaced by: “*Among genes present on the Hsa21, the Dual-specificity Tyrosine-(Y)-phosphorylation-Regulated Kinase 1A (DYRK1A), the mammalian homologue of the Drosophila minibrain (mnb) gene that is essential for normal neurogenesis, is a target for improvement of DS cognition*”

3. Please use similar axes for similar tests. It would be useful to have Figures 3I and 3J on the same axes for comparison.

YH> Figure 3I (now figure 4I) changed to have the same scale on the Y axis. Figures 3E and 3F (now figures 4E and 4F)were also changed to have the same scale between the two.

4. Some of the references in the manuscript are not defined and still have notes from the authors in them (Lines 292, 316, 429, 434, 458, 469, 483, 491, for example).

YH> Sorry for the references, they have been included

5. Person first terminology should be used and the term “patients” should be avoided (line 503). Perhaps “Individuals with DS?”

YH> We changed the “patients” to “persons” in page 6 line 133 and to “Individuals with DS “ in page 22, line 507.

**Reviewer #2:** In this study, Brault et al. use conditional knockout technology to study the role of Dyrk1a gene dosage in the function of excitatory glutamatergic neurons in cognition. Using Camk2a-Cre to delete floxed Dyrk1a alleles in glutamatergic neurons of wild type mice or mice modeling trisomy 21, they examine locomotor activity, working and long-term memory, and susceptibility to epileptogenesis. They further examine effects of gene dosage on gene expression. The main conclusion is that Dyrk1a dosage impacts long term memory, possibly through modulation of long-trem synaptic plasticity. The role of the gene in glutamatergic neurons has not been investigated previously, though recent studies have suggested it might be important in synaptic properties of these neurons.

Dyrk1A excess gene dosage is implicated in neurological deficits in Down Syndrome, and loss of this gene is associated with a severe autism syndrome. Because the gene is involved in multiple processes in brain development and function, it is difficult to pinpoint specific loci of its action via conventional gene knockout. This study adds to our knowledge concerning the postnatal actions of this gene. There are a number of areas in which it is difficult to draw a clear relationship between the effects of gene dosage, the anatomical substrates, and the alterations in gene expression, and the clarity of the study would be improved by less extrapolation of the data and speculation regarding these relationships.

1. Page 71 DS is “the first identified genetic cause of mental retardation”

YH> We changed to “Down syndrome (DS; Trisomy 21), is the leading genetic cause of intellectual disability”.

2. Results line 142 and following: did the authors investigate in detail the degree of specificity of Camk2a driven deletion for glutamatergic neurons?

YH> We have done in our laboratory the characterisation of the line with another gene inactivation. Cre-deletion specificity was assessed in Dubos et al. 2015 (10.1093/hmg/ddv380) using the same Camk2Cre mouse to delete *Atp6ap2* which we showed to be expressed in all the Camk2a-positive cells. We showed that Cre deletion occurred in around 57% of the Camk2Cre+ cells of the cortex and in about 84% of the Camk2Cre+ cells in the hippocampus. Saying that, the Camk2a-Cre delete mouse that we used is a standard deleter mouse that has been commonly used:

- Dragatsi and Zeitlin, 2000. CaMKIIa-cre Transgene Expression and Recombination Patterns in the Mouse Brain
- Mantamadiotis et al. 2002. Disruption of CREB function in brain leads to neurodegeneration
- Marsicano, G., Goodenough, S., Monory, K., Hermann, H., Eder, M.,Cannich, A., Azad, S.C., Cascio, M.G., Gutie´ rrez, S.O., van der Stelt, M., et al. (2003). CB1 cannabinoid receptors and on-demand defense against excitotoxicity. Science 302, 84–88.

We added this information to our manuscript (Result, paragraph 1):

Page 7, lines 151-155: “*This transgene starts to express the Cre recombinase at three weeks of age in the cortex and hippocampus, when most neural circuits are already formed, as described previously [28, 31, 32].* *Our laboratory has previously shown that Cre deletion occurs in around 60% of the Camk2Cre-positive cells in the cortex and in around 80% of the Camk2Cre-positive cells of the hippocampus [33]*.“

3. Line 160-how might the changes in cell morphology or tissue organization in the cortex have affected the readouts of behavioral tests?

YH> We agree that change in cell morphology or tissue organization in the cortex can affect the circuits implicating cortical neurons and changing the input or output of the different pathways. We discussed this point in the discussion:

- Page 20, line 456: “*Beside its role in the perception and processing of sensory information, the somatosensory cortex is also implicated in the integration of high order information and participates in linking perception of socially relevant stimuli to motivation, emotion and perception [55], features that are strongly perturbated in ASD.”*
- Page 21, line 473: “…it was shown that context freezing one day after conditioning partly occurs through reactivation of neuronal ensembles in the primary sensory cortex.”

What neurons (or other cell types) were affected?

YH> Although we see a decrease in cortical thickness, we do not observe a decreased in the number of cells within the cortex (counting bodies of cells stained in purple with luxol fast blue) but an increase in cell density, suggesting that there might be neuronal atrophy that would explain the decrease in cortical thickness. As asked by reviewer 3, we made a NeuN staining to label glutamatergic neurons to confirm that neuronal density is indeed augmented in the SSC of *Dyrk1a^C/C^* mice.

As discussed, (Page 20 line 445), “*In comparison, Dyrk1a^+/-^ mice from a mixed B6/129OLA genetic background showed 30% brain reduction and the cortical size reduction was also associated with increased cell density, as described in Dyrk1a^+/-^ [34][26], suggesting a reduction of neuronal processes as observed in the neocortex of Dyrk1a^+/-^ mice [19, 26, 54].*”

What behavioral changes may be said with confidence to relate solely to deletion in hippocampal neurons?

YH> We know from the literature that the Novel object recognition requires a network of medial-temporal lobe regions including the hippocampus and the perirhinal, parahippocampal, entorhinal and insular cortices (Cohen and Stackman 2015 ). Encoding of contextual fear memory requires the hippocampal-amygdala circuit together with frontal cortical region (Izquierdo et al. 2016).

We added to the discussion:

Page 20, line 469: *“Encoding of contextual fear conditioning memory requires the hippocampal-amygdala circuit together with frontal cortices region [57]Deficit in contextual fear behaviour is often attributed to a defective hippocampal-to-basolateral amygdala transmission as a result of either a deficit in glutamatergic projections or deficit in excitatory activity [58], although* *it was shown that context freezing one day after conditioning partly occurs through reactivation of neuronal ensembles in the primary sensory cortex [59].”*

Page 23, line 511: “*Novel object recognition requires a network of medial-temporal lobe regions including the hippocampus and the perirhinal-parahippocampal-entothinal and insular cortices [69].”*

4. Line 251 and following: scRNA-seq might have been quite informative here.

YH> We totally agree that scRNA-seq analysis, and even more Single-Nuclei RNaseq on neurons would have been more informative than the bulk RNAseq analysis that we performed. Nevertheless, when we started the experiments, Single-Nuclei RNaseq was not available. We aim at doing this experiment in a follow-up study.

5. Line 256 following: what exactly is going on with the oligodendrocytes

YH> As detailed in lines 265-272, the up-regulated genes were enriched in oligodendrocyte-specific genes, but we could did not see any increased density in oligodendrocytes in the corpus callosum. Using the list of up-regulated genes that are oligodendrocyte-specific, we searched if this list was enriched for specific biological processes with FunRich (<http://www.funrich.org/>) and found that 8 of the 27 genes were implicated in cell communication and signal transduction. Thus, we speculate that impact of *Dyrk1a* inactivation in glutamatergic neurons has a secondary consequence on communication between depleted neurons and the oligodendrocytes.

We added this information in our manuscript as a supplementary table S3 and integrated this information in the result:

Page 13, line 304: “*Using the list of up-regulated genes that are oligodendrocyte-specific, we searched if this list was enriched for specific biological processes with FunRich (*[*http://funrich.org*](http://funrich.org)*) and found that eight of the twenty-seven listed genes were implicated in cell communication and signal transduction, suggesting that Dyrk1a inactivation in glutamatergic neurons might have a secondary impact on communication between those neurons and oligodendrocytes*.”

6. Line 273 these genes were upregulated in what cell type? The authors might at least perform immunostaining or RNA-Scope for the more important ones?

YH> Although our RNAseq analysis was made on RNA transcripts from all cell populations of the hippocampus, the hippocampus contains a majority of glutamatergic neurons (interneurons represent only ~10–15% of the total neuronal population) and, as Dyrk1a deletion was done in glutamatergic neurons, we can speculate that immediate-early genes are most probably down-regulated in glutamatergic neurons, as a cell-autonomous consequence of Dyrk1a inactivation in those neurons. But we cannot rule out that there is also an indirect effect of this inactivation on immediate early gene expression in GABAergic neurons as immediate early genes are expressed in both cell populations. We agree that immunostaining or RNA-Scope could be informative but we doubt that they are quantitative enough to answer the question.

7. Line 316-remove “Duchon and Herault HMG?

YH> We removed this and added the full reference

8. Did the authors do proteomics in Dyrk1aC/C mice to compare with RNA-seq?

YH> No we did not do proteomics in Dyrk1aC/C mice.

9. Line 391-were blocking peptides used to check specificity of pulldown?

YH> No but we used several antibodies and we never get cross-contaminants.

10. Line 397 and following: the Discussion is far too long; statements that are largely speculative and center around the many remaining unknowns could be removed or truncated to help the reader focus on the strong conclusions of the study. For example: role of IEG genes, normalization of gene copy number and memory, LTP.

YH> We completely re-wrote and shortened (2 pages removed) the discussion to fit with the demands of the reviewers. We first discussed the impact of the deletion of Dyrk1a in glutamatergic neurons and compared our results with previous works on *Dyrk1a^+/-^* and *Dyrk1a^EmxCre/+^* animals as demanded by reviewers 3 and 4. We focused then on the role of DYRK1A in glutamatergic neurons in explicit long-term memory and removed the more speculative statements.

11. Line 434-insert numbered reference Duchon et al.

YH> The reference has been updated

12. Line 573 following: clarify the genetic backgrounds of the various mouse mutants actually studied.

YH> A supplementary table (7) was added to precisely show the genetic background for the different mouse lines as stated for the reviewer 1.

**Reviewer #3:** The manuscript by Brault et al. focusses on the role of DYRK1A kinase in the glutamatergic neurons of the cerebral cortex and hippocampus. *DYRK1A* gene is on chromosome 21 and it is widely expressed in the developing and mature brain. The authors generated a conditional KO mouse designed for the elimination of DYRK1A in the glutamatergic neurons of the postnatal forebrain and assessed the impact of this conditional mutation on brain morphology, cognition and other behaviours associated to MRD7, a rare intellectual disability syndrome caused by loss-of function mutations in the *DYRK1A* gene. In this conditional KOs, they found alterations in the cytoarchitecture of the neocortex and specific cognitive functions. Moreover, they show RNA-seq data suggesting that the lack of DYRK1A in glutamatergic neurons affects the expression of genes involved in synaptic activity and transcription. Using the new conditional *Dyrk1a* mutant they normalize *Dyrk1a* gene-dosage in the glutamatergic neurons of the Dp1Yey mouse, a trisomic model for Down syndrome. Then, they performed several behavioural tests in disomic and trisomic mice with or without the conditional *Dyrk1a* mutation in heterozygosity. Their results show that both haploinsufficiency of *Dyrk1a* and trisomy of Dyrk1a in postnatal glutamatergic neurons have detrimental effects on explicit memory. Finally, they present proteome data indicating that DYRK1A may regulate the activity of glutamatergic synapses.

The study is of interest and some of the results are well explained and are convincing, but others have raised some concerns that the authors need to address by providing new information and experimental data.

Major issues:

1. The quality of the brain images in Figure 1 need to be improved, especially in panel E. The authors should include information in the figure and the legend regarding the area of the neocortex, the age of the animal and, in panel E, the number of animals analysed.

DYRK1A staining in the neocortex is mostly associated to the neuropile (Martí et al., Brain Res 2003), but this do not seem to be the case in the images of the cortex shown in panels A and E, where DYRK1A labelling in the soma of CAMK2+ cells is high. Is DYRK1A staining similar in other cortical regions (e.g. somatosentory and motor cortex)? Did the authors perform control stainings with secondary antibodies?

YH> Figure 1A has been improved as also asked by reviewer 1 and we added a supplementary Figure 1. We have added the DAPI staining to the images to better see that DYRK1A and CAMK2A signals are mainly perinuclear. Our immunostaining for DYRK1A is like that shown by Altafaj et al. 2013, figure 3B (PMID: 23220201).

To improve image quality of fig. 1E, we changed the images of panel E, making higher resolution images and changing DYRK1A signal in grey.

We added the information asked in the figure legend:

*“Supplementary figure 1 DYRK1A (red) co-localizes with CAMK2A (green) in the glutamatergic pyramidal neurons of the CA1-3, the granular neurons of the dentate gyrus (DG) and in the cortex (photo at the level of the auditory cortex) of a 3-month-old wild-type control mouse.”*

*“Figure 1(E) DYRK1A immunohistochemistry of coronal brain sections around Bregma -1.5 mm (Paxinos adult mouse brain atlas, Franklin and Paxinos, 1997) at the level of the hippocampus and of the cortex area above the hippocampus from a control and a Dyrk1a^C/C^ mouse (2-month-old males).”*

We observed similar Dyrk1a staining in all the cortical regions.

We did perform control staining with secondary antibodies. We added a sentence in Material and Methods.

Cre-recombinase in the Tg(CamK2-Cre)4 is wildly expressed in the postnatal forebrain. The authors should emphasise this point at the beginning of the Results. Do the authors know the onset of Cre-recombinase expression in the hippocampus and neocortex of cKO mice? This information is relevant for the correct interpretation of the results and should be added in the manuscript.

YH> We agree with the reviewer. This was indeed an important point that we forgot to mention in our manuscript. We have added this information in the introduction:

*“We selected the Tg(Camk2a-Cre) transgene to target the Cre recombinase in* ***postnatal*** *glutamatergic neurons within the forebrain”*  and in the results:

*“We wanted to better understand the function of DYRK1A in adult glutamatergic neurons, circumventing the effect of Dyrk1a on brain development.”….”This transgene starts to express the Cre recombinase at three weeks of age in the cortex and hippocampus, when most neural circuits are already formed.”*

2. Based on morphological alterations of Dyrk1a KO neocortices (thickness and total cell densities; Figure 2) and the similarities of these alterations with those previously observed in Dyrk1a+/-, the authors suggest that DYRK1A has a direct role on late morphological differentiation of glutamatergic neurons. This is an interesting possibility that could be tested by looking in the conditional Dyrk1a KO the morphology of projection cortical neurons after synaptic pruning. Regarding the data shown in Figure 2. Cell density in the image of the Dyrk1a KO somatosensory cortex (panel F) seems to be decreased rather than increased, in particular in layers II-III. Is this a representative image? The authors should provide magnifications of the images to better illustrate the differences in cell numbers and cell densities between genotypes. In addition, they should label the glutamatergic neurons (using for instance the neuronal marker NeuN) of the adult cerebral cortex to confirm that neuronal densities are indeed augmented in the SSC of Dyrk1a KOs.

YH> We agree and we selected better images with higher magnification for panel F. We made NeuN immunostaining and counted NeuN+ neurons. We added a new figure (Figure 3) with representative images of luxol-stained and NeuN-labeled sections with inset showing higher magnification of the area within the SSC were cell counts were done. We confirmed that neuronal densities were augmented in the SSC of DYRK1A K0s.

Page 9, line 194: “*We confirmed that neuronal density was indeed increased in the SSC of Dyrk1a^C/C^ mice by labelling neurons with the NeuN marker and counting NeuN-positive cells (Figure 3B, E).”*

3. Differences between the transcriptome of controls and Dyrk1a KOs are based on RNA-seq data obtained from only two animals per genotype. The authors should definitely perform RT-PCRs to confirm the differential expression of immediate early genes and genes involved in neurotransmission in additional animals. Otherwise, this data should be removed.

YH> We agree with the reviewer that there is a limitation here. The small number of individuals used for this analysis can only unravel highly differentially expressed genes between the two conditions. We already used this type of comparison with 2-3 samples per condition in other models (Dubos et al 2015; Iacono et al. 2017; Dubos et al 2018) in which we used hypergeometric test and Bonferroni correction. Moreover, we found some misregulated genes that were already described deregulated in DS models (immediate early genes Npas4, Arc, c-fos and Fosb: Braudeau et al. 2011; Duchon et al. 2021; genes coding for proteins involved in synaptic vesicle fusion: Duchon et al. 2021) with more individuals per groups, and which were confirmed by other techniques.

We added those informations:

Page 12, line 282: *“Analysis of the RNA-seq exon reads was performed using hypergeometric test and Bonferroni correction (DEseq algorithm, P<0.025) as previously published [33, 42, 43] and identified 297 up-regulated and 257 down-regulated genes in Dyrk1a^C/C^ compared with controls*”

Page 21, line 484: *“Interestingly, we and our collaborators observed a decreased in the expression of some of these IEGs (Npas4, Arc, c-Fos and Fosb) in the hippocampi of Tg(Dyrk1a) and of the trisomic mouse models Dp1Rhr and Ts65Dn [45, 64].”*

Page25, line 566: *“Moreover, DYRK1A was found to phosphorylate MUNC18-1 [88], which interacts with the SNARE complex protein Syntaxin 1A, whose transcripts are also decreased in the hippocampus of Dyrk1a^C/C^ mice and which was found to be one of the six hubs connecting the major subnetwork biological cascades found deregulated in DS models [45].”*

4. The results of the proteome analysis are interesting but are too short to conclude that DYRK1A in glutamatergic neurons regulates synaptic activity in a gene-dosage dependent manner. The authors should perform additional experiments (measure LTP or at least check by immunoblotting the levels of several of the putative differentially expressed proteins involved in synaptic activity, including CAMK2) to support this conclusion.

YH> We totally agree that performing measurement of LTP activity to show *Dyrk1a*-dosage dependent impact on synaptic activity would have been the experiment to validate our molecular and behavioral observations. We indeed already mentioned this in the discussion:

Page 23, line 530: *“As Dp1Yey mice show similar hippocampal LTP deficit [74], it would be interesting to see if Dyrk1a normalisation in the glutamatergic neurons could restore LTP in Dp1Yey/Dyrk1a^C/+^ mice.”*

We however do not have the expertise to perform this kind of experiment in our laboratory and our attempt to find a collaborator to do such an analysis was hampered by the difficulty of potential collaborators to produce animals with such a complex genotype (triple mutant: Dp1Yey, Camk2Cre, Dyrk1a cKO) or to receive, host and analyze so many animals (4 genotypes). We think that this publication will ease a future collaboration to resolve this point.

Regarding the variations of protein amounts observed in the proteomic analysis, they are small and will be very difficult to detect by Western blot analysis, especially for some of the interesting proteins that have high molecular weight.

The co-IPs shown in figure 5 suggest that DYRK1A and CAMK2 are part of the same protein complex. Is SYN1 also part of this complex?

YH> We agree with the reviewer that probably SYN1 can be part of the complex in the presynapse. We already discussed the participation of DYRK1A to a CAMK2-SYN1 complex in the presynaptic compartment in our previous work by Nguyen et al (2017) as stated in Page 18, Line 419: “*Previous work from our laboratory already demonstrated a role of DYRK1A at the presynapse by showing interaction of DYRK1A with SYN1, a neuronal phosphoprotein associating with the cytoplasmic surface of the presynaptic vesicles and tethering them to the actin cytoskeleton [47, 48]”*

On the other hand, DYRK1A seems to interact with proteins of postsynaptic densities. Is there any evidence that DYRK1A is in the presynaptic part and PSD of excitatory synapses?

YH> There are many evidence for localisation as well as a role of DYRK1A at the presynapse. Presence of Dyrk1a/Mnb was described in the presynaptic terminal of motor neurons in the neuromuscular junction where it was required for normal synaptic growth and vesicle endocytosis (Arque et al. 2013; Chen et al. 2014) DYRK1A regulates clathrin-mediated vesicle endocytosis via phosphorylation of the phosphatase synaptojanin I, the GTPase dynamin I, the scaffold protein amphyphysin I and the clathrin assembly proteins AP180, MAP1A, MAP2 and α- and β-adaptins (Adayev et al. 2006; Huang et al. 2004; Chen et al. 2014; Geng et al. 2016; Murakami et al. 2006, 2009, 2012). Overexpression of DYRK1A in neurons results in the inhibition of endocytosis due to defects in clathrin-mediated endocytosis (Kim et al. 2010). Evidence for a role of DYRK1A at the presynapse comes from its localisation and its tight coupling to the endocytic machinery (Arque et al. 2013; Chen et al. 2014). DYRK1A was found to interact with and/or phosphorylate the presynaptic proteins MUNC18-1, a central regulator of SNARE-mediated exocytosis (Park et al. 2012b), the α-synuclein which chaperones the SNARE-complex assembly (Burré, 2015) and synapsin I involved in the maintenance of the reserve of the synaptic vesicle pool at the presynapse (Nguyen et al. 2018). Interestingly, in an in-depth transcriptomic analysis of the hippocampus of six DS mouse models, Duchon and collaborators highlighted DYRK1A and SNARE as part of six central hubs connecting six major biological subnetworks deregulated in those models (Duchon et al. 2021).

We mentioned some of these points in our discussion:

Page 25, line 562: “*Dyrk1a^C/C^ transcriptomic analysis also revealed a down-regulation of genes for proteins involved in presynaptic vesicle cycle, implicating DYRK1A in neurotransmitter release. Among the deregulated genes, we found Amphiphysin (Amph), which its protein is a known target of DYRK1A [87], and Synapsin 2 (Syn2), which paralog SYNAPSIN 1 was found to be hyperphosphorylated by DYRK1A overexpression in TgDyrk1a mice [51].* *Moreover, DYRK1A was found to phosphorylate MUNC18-1 [88], which interacts with the SNARE complex protein Syntaxin 1A, whose transcripts are also decreased in the hippocampus of Dyrk1a^C/C^ mice and which was found to be one of the six hubs connecting the major subnetwork biological cascades found deregulated in DS models [45]. Our results together with others [87, 89, 90] point at a role of DYRK1A in the glutamatergic presynapse in the control of neurotransmitter release through synaptic vesicles exocytosis and vesicles recycling processes.”*

Interaction of DYR1KA with NR2B and PSD95, two PSD proteins, is clearly in favour of the presence of DYRK1A in the post-synapse. Moreover, there are in the literature additional evidence of a role of Dyrk1a at the post synapse. DYRK1A was found to phosphorylate the NMDAR subunit NR2A on its C-terminal, modifying its biophysical properties in favour of channel opening (Grau et al. 2014). Altafaj and collaborators also reported an increased NR2A expression in the brain of TgDyrk1a mice together with higher calcium uptake after NMDA stimulation (Altafaj et al. 2008). They also reported decreased internalization of the NR2A-containing NMDAR resulting in their accumulation at the surface of the post-synapse. DYRK1A was finally found to interact with and phosphorylate SPROUTY, a protein involved in receptor trafficking (Aranda et al. 2008).

We have added this information in the discussion:

Page 24, line 545:

“*Previous work done in our laboratory found several proteins from the PSD that were hyperphosphorylated in mice with three copies of Dyrk1a (TgDyrk1a) [34] and dephosphorylated by TgDyrk1a mouse treatment with a DYRK1A inhibitor [49]. Among those proteins, the NR2B is a subunit of the glutamatergic postsynaptic NMDA receptor which play a pivotal role in excitatory synaptic transmission. This result was validated by our Co-IP experiments showing an interaction between DYRK1A and NR2B. NR2B subunits are expressed in the neocortex and hippocampus [74-76]. NMDA receptors in the mature hippocampus consist of two NR1 subunits associated with either two NR2A, two NR2B or one of each subunits [77, 78] and different forms of synaptic plasticity have been associated to different types of NMDA receptors [79-81]. Hence, in addition to its interaction with the NR1/NR2A-type of receptors [82], we also point out an association with NR1/NR2B receptors. Moreover, interaction between DYRK1A and the PSD proteins PSD95, CAMK2 and SYNGAP, detected by coIP, strongly suggests a role of DYRK1A at the glutamatergic postsynapse. In addition, absence of the GLUR1 subunit of the AMPA receptor in the IP of DYRK1A indicates that DYRK1A interact most specifically with the NMDA-PSD complex. Altogether, this strongly suggests an implication of DYRK1A at the glutamatergic post-synapse, somehow supporting its involvement in long-term memory formation*.”

The title of this section “Proteomic analysis confirms…..” is an overstatement, I suggest to change it.

YH> We agree and replaced by: “Proteomic analysis suggests an impact of *Dyrk1a* gene dosage on synaptic activity.”

Minor issues:

1. According to ORPHANET, the name for MRD7 (Mental Retardation Dominant 7) is DYRK1A-intellectual disability syndrome. This syndrome is also named “DYRK1A haploinsufficiency syndrome”. I suggest to use one of these two names instead of MRD7. The authors should definitely indicate the ORPHA numbers of the syndrome. Additionally, they should add in the Introduction information regarding the type of mutations that are causing the syndrome and, in References, two papers by van Bon et al., one describing the first case with an intragenic deletion in *DYRK1A* genes (Clin Genet. 2011) and, the second one, the main clinical features associated to the syndrome (Molecular Psychiatry 2015).

YH> The name MRD7 is still use at the NCBI <https://www.ncbi.nlm.nih.gov/gtr/conditions/C3279839/> and in the OMIN Database entry 614104 https://www.omim.org/entry/614104 . Nevertheless, we added into the introduction the two descriptions for a broader audience and included the two reference papers proposed by the reviewer: Page 4, line 75:

*“Among genes present on the Hsa21, the Dual-specificity Tyrosine-(Y)-phosphorylation-Regulated Kinase 1A (DYRK1A), the mammalian homologue of the Drosophila minibrain (mnb) gene that is essential for normal neurogenesis [1,2], is a target for improvement of DS cognition [3]. In addition, 21q22.13-22.2 microdeletion syndrome associated to DYRK1A* ***(ORPHA:268261) and*** *intellectual deficit due to* ***mutations in DYRK1A*** *(****ORPHA:464311; also known as mental retardation, autosomal dominant disease 7: MRD7; OMIM #614104) show neurodevelopmental anomalies****[4-9], making this gene a critical dosage-sensitive gene for cognitive phenotypes.”*

2. There is information in the Introduction regarding the molecular functions of DYRK1A that is not relevant for the paper and should be eliminated or at least shortened (paragraph lines 79 to 88). Instead, the description of the neurological deficits and behaviours associated to MRD7 is too short.

YH> We added some information regarding neurological deficits and behavior associated to MRD7 in the introduction and removed the information about molecular function.

3. Haploinsufficient Dyrk1a+/- mice show epileptic activity and susceptibility to hyperthermia-induced seizures as well as deficits in social communication (Raveau et al. Neurobiol Dis 2018 and Arranz et al. Neurobiol Dis. 2019). The authors should consider these two works for the interpretation and discussion of their data (deficits in social behaviour and lack of response to PTZ in Dyrk1a KOs) in the context of MRD7.

YH> We added this to the discussion: pages 19-20, lines 456-465:

*“**Beside its role in the perception and processing of sensory information, the somatosensory cortex is also implicated in the integration of high order information and participates in linking perception of socially relevant stimuli to motivation, emotion and perception [53], features that are strongly perturbated in ASD. We found that Dyrk1a^C/C^ mice spent less time in social contact than control mice, a phenotype also observed in Dyrk1a^+/-^ and Dyrk1a^Emx1/+^ animals [51, 54, 55], suggesting a role for DYRK1A in cortical glutamatergic neurons in ASD-like deficits observed in persons with DYRK1A mutations. However, postnatal inactivation of Dyrk1a in glutamatergic neurons was not sufficient to trigger susceptibility to epileptic activity as observed in Dyrk1a^+/-^ mice [54, 56], suggesting that absence of DYRK1A in glutamatergic neurons is not responsible for this phenotype.”*

4. Please indicate the age of the animals used in each experiment/behavioural test in the figure and/or the figure legend. In Fig. 3, also indicate the sex of the animals tested and for clarity, use the same colour code than in Fig. 4.

YH> The age of the animals was added either to the figure legends or in Material and Methods (for histological and molecular analyses). For behavior experiments, a supplementary table 10 was added with the age, sexes and number of animals. There is no color code for animals in figure 3 because we only used males.

**Reviewer #4:** This manuscript by Brault et al. reports findings from manipulating Dyrk1a gene dosage in glutamatergic neurons. Homozygous and heterozygous conditional mutants for Dyrk1a using CamK2-Cre, and this was combined with Dp1Yey trisomy model. Effects of Dyrk1a deletion in glutamatergic neurons on brain morphology and behavior were assessed, revealing deficits in a variety of behavioral domains. Behavioral consequences of reducing Dyrk1a dosage in a Dp1Yey background was also tested. Proteomic analysis of differentially abundant proteins in mutant backgrounds identified synaptic proteins that are candidate interactors with Dyrk1a. Overall, this manuscript reports multiple interesting molecular, anatomical and behavioral effets of CamK2-Cre-mediated deletion of Dyrk1a. However, these observations are not tied together into a coherent story and there are several areas where the manuscript could be improved, listed below.

MAJOR CRITIQUES
- A major weakness of this study is that there are no analyses at the level of neuronal structure or function that could connect the observations of abnormal abundance of key neuronal proteins, altered brain morphology and density of cortical neurons with abnormal behavior in conditional Dyrk1a mutants. This is a major gap in the study design. For example, a direct measure of glutamatergic neuronal morphology would corroborate the interesting finding of altered density.

YH> Neuronal morphological changes due to reduced *Dyrk1a* dosage have been already described. The decreased cortex size and increased density of cortical neurons with no change in cell number was already seen in *Dyrk1a^+/-^* animals (Guedj et al. 2012) and our results with the Camk2Cre conditional knockout animal reveal the role of DYRK1A on post-natal neuronal morphogenesis. This phenotype was already previously linked to a reduction of neuronal processes in the cortex of *Dyrk1a^+/-^* mice (Arqué et al. 2008, Benavides-Piccione et al. 2005; Dang et al. 2018). We mentioned this in the discussion(page 20, lane 445):

*“In comparison, Dyrk1a^+/-^ mice from a mixed B6/129OLA genetic background showed 30% brain reduction and the cortical size reduction was also associated with increased cell density, as described in Dyrk1a^+/-^ [34][26], suggesting a reduction of neuronal processes as observed in the neocortex of Dyrk1a^+/-^ mice [19, 26, 54]..”*

We do not agree that showing a change in neuronal morphology will help to link cortical phenotype to specific abnormal behaviors, that are complex phenotypes related to complex neuronal pathways implicating several brain structures. We added this point in our discussion:

- page 19, line 456: “*Beside its role in the perception and processing of sensory information, the somatosensory cortex is also implicated in the integration of high order information and participates in linking perception of socially relevant stimuli to motivation, emotion and perception [55], features that are strongly perturbated in ASD.”*
- Page 20, line 473: “it was shown that context freezing one day after conditioning partly occurs through reactivation of neuronal ensembles in the primary sensory cortex.”

- The logic of using CamK2-Cre to delete Dyrk1a should be better explained. Why was this line, which will delete Dyrk1a later in the development of glutamatergic neurons, used? There may be a compelling reason, e.g. circumventing early developmental functions of Dyrk1a, but this should be explained.

YH> This was indeed an important point that we forgot to mention in our manuscript. We have added this information in the introduction:

Page 6, line 125: *“We selected the Tg(Camk2a-Cre) transgene to target the Cre recombinase in* ***postnatal*** *glutamatergic neurons within the forebrain”*  And in the results (page 7, line 145):

*“We wanted to better understand the function of DYRK1A in adult glutamatergic neurons, circumventing the effect of Dyrk1a on brain development.”….”* *This transgene starts to express the Cre recombinase at three weeks of age in the cortex and hippocampus, when most neural circuits are already formed, as described previously [28, 31, 32].”*

- Relatedly, another study (<https://doi.org/10.1016/j.biopsych.2021.01.012>) has recently been published that deleted Dyrk1a using Emx1-Cre, resulting in an earlier developmental loss of Dyrk1a function in glutamatergic neurons. While it is understandable that this paper was not cited given the timing of publication, the findings of the two studies (i.e. earlier and later deletion of Dyrk1a in glutamatergic neurons) are complementary and should be discussed.

YH> Indeed, this paper was not published when we submitted our manuscript. We have included it in our discussion:

- Page 20, line 441: “*Contrary to Dyrk1a inactivation in cortical progenitor cells and glutamatergic postmitotic neurons leading to a lack of corpus callosum and lateral cortex with the death of the animal after birth [53], inactivation of Dyrk1a in postnatal glutamatergic neurons results in a milder phenotype. Dyrk1a^C/C^ mice are viable and present a mild microcephaly with a 10% brain weight reduction and a principal impact on cortical thickness. “*

- Page 20, line 449: “*Surprisingly, mice with heterozygous inactivation of Dyrk1a in developing excitatory neurons of the cortex (Dyrk1a^Emx1/+^) presented decreased cortical thickness and increased cell density due to smaller excitatory neurons only at birth, while adult microcephaly was associated to fewer neurons due to postnatal cell death due to increased apoptosis [53]. This suggests that microcephaly observed in MRD7 results from different impacts of DYRK1A on brain neurogenesis during embryonic and postnatal development, with Dyrk1a^C/C^ brain revealing the impact of DYRK1A on postnatal neuronal morphogenesis.”*

- The description of the RNAseq experiment could be written more clearly and succinctly.

YH> We agree, and we shortened the description of the RNAseq experiment (lane 710)

- For reporting of behavioral results, males and females are pooled and represented with different colored dots in Figure 4, while this is not done in Figure 3. Why are the results reported differently between these two figures? Why where sexes pooled instead of analyzed separately?

YH> Mice are not colored by sex in other figures because only males were used for *Dyrk1a^C/C^* behavioral analysis (information added to Material and Methods). As stated in Material and Methods, due to the difficulty to obtain Dp1Yey*; Dyrk1a^C/+^* mice (3 alleles for the crossing and subfertility of the Dp1Yey line), both males and females were pooled for the analysis to have enough animals for statistical analysis.

We were confident in pooling males and females for the following reasons:

- In our behavioural analyses, males and females are well distributed, which does not suggest any sex difference
- There are no data suggesting a sex effect in ID observed in DS persons.
- Faizi and collaborators (Faizi et al. 2011) underwent behavioural analysis on both males and females Ts65Dn (locomotor activity, spontaneous alternation, water maze, and contextual fear conditioning) and showed no difference between males and females.

  MINOR CRITIQUES
  - Lines 103-105 require a reference.

YH> We Added: Arque et al., 2008; Benavides-Piccione et al., 2005; Fotaki et al., 2002

- The amount of hippocampal GAPDH in figure 1H appears different between control and mutant. Is this due to different total protein concentration between the two samples? Or is there actually a change in GAPDH levels in mutants?

YH>There is indeed a slight decrease of GAPDH in the mutant, although the same quantity of proteins was loaded onto the gel. But the change in amount of GAPDH between the 2 genotypes is not significant (see table below) and does not change the interpretation of the result, showing that the amount of Dyrk1a protein is indeed decreased in the mutant mice.

**Have all data underlying the figures and results presented in the manuscript been provided?**
Large-scale datasets should be made available via a public repository as described in the *PLOS Genetics* [data availability policy](http://journals.plos.org/plosgenetics/s/data-availability), and numerical data that underlies graphs or summary statistics should be provided in spreadsheet form as supporting information.

Reviewer #1: Yes

Reviewer #2: Yes

Reviewer #3: Yes

Reviewer #4: Yes

PLOS authors have the option to publish the peer review history of their article ([what does this mean?](https://journals.plos.org/plosgenetics/s/editorial-and-peer-review-process#loc-peer-review-history)). If published, this will include your full peer review and any attached files.


If you choose “no”, your identity will remain anonymous but your review may still be made public.


**Do you want your identity to be public for this peer review?** For information about this choice, including consent withdrawal, please see our [Privacy Policy](https://www.plos.org/privacy-policy).

Reviewer #1: No

Reviewer #2: No

Reviewer #3: No

Reviewer #4: No

*In compliance with data protection regulations, you may request that we remove your personal registration details at any time.* [*(Remove my information/details)*](https://www.editorialmanager.com/pgenetics/login.asp?a=r)*. Please contact the publication office if you have any questions.*
